# Supplementary material for: Δ133p53β isoform pro-invasive activity is regulated through an aggregation-dependent mechanism in cancer cells
Source: Nat Commun. 2021 Sep 15;12:5463. doi: 10.1038/s41467-021-25550-2 (PMC8443592; doi:10.1038/s41467-021-25550-2)
Supplement: Supplementary file 1 — Supplementary Information [file 41467_2021_25550_MOESM1_ESM.pdf]

Supplementary Information

Δ133p53β isoform pro-invasive activity is regulated through an aggregation-dependent mechanism in cancer cells.

Nikola Arsic [1,2], Tania Slatter[3], Gilles Gadea[4], Etienne Villain[1,2], Aurelie Fournet[5,2], Marina Kazantseva[3], Frédéric Allemand[5,2], Nathalie Sibille[5,2], Martial Seveno[6,2], Sylvain de Rossi[7], Sunali Mehta[3], Serge Urbach[8,2], Jean-Christophe Bourdon[9], Pau Bernado[5,2], Andrey V Kajava[1,2,10], Antony Braithwaite[3] and Pierre Roux[1,2]

1. Université de Montpellier, Centre de Recherche en Biologie Cellulaire de Montpellier (CRBM), CNRS, UMR 5237, Montpellier, France
2. Université de Montpellier, Montpellier, France
3. Department of Pathology, University of Otago, Dunedin, New Zealand
4. Université de la Réunion, Unité Mixte 134 Processus Infectieux en Milieu Insulaire Tropical, INSERM Unité 1187, CNRS Unité Mixte de Recherche 9192, IRD Unité Mixte de Recherche 249. Plateforme Technologique CYROI, Sainte Clotilde, France.
5. Centre de Biochimie Structurale (CBS), INSERM, CNRS, 29 rue de Navacelles, Montpellier, France
6. BioCampus Montpellier, CNRS, INSERM, Montpellier, France.
7. MRI, UMS BioCampus Montpellier, CNRS, INSERM, Université de Montpellier, Montpellier, France.
8. IGF, CNRS, INSERM, Montpellier, France.
9. Dundee Cancer Centre, University of Dundee, Ninewells Hospital and Medical School, Dundee, United Kingdom.
10. Institut de Biologie Computationnelle, Montpellier, France

To whom correspondence should be addressed: pierre.roux@crbm.cnrs.fr

Supplementary figure 1

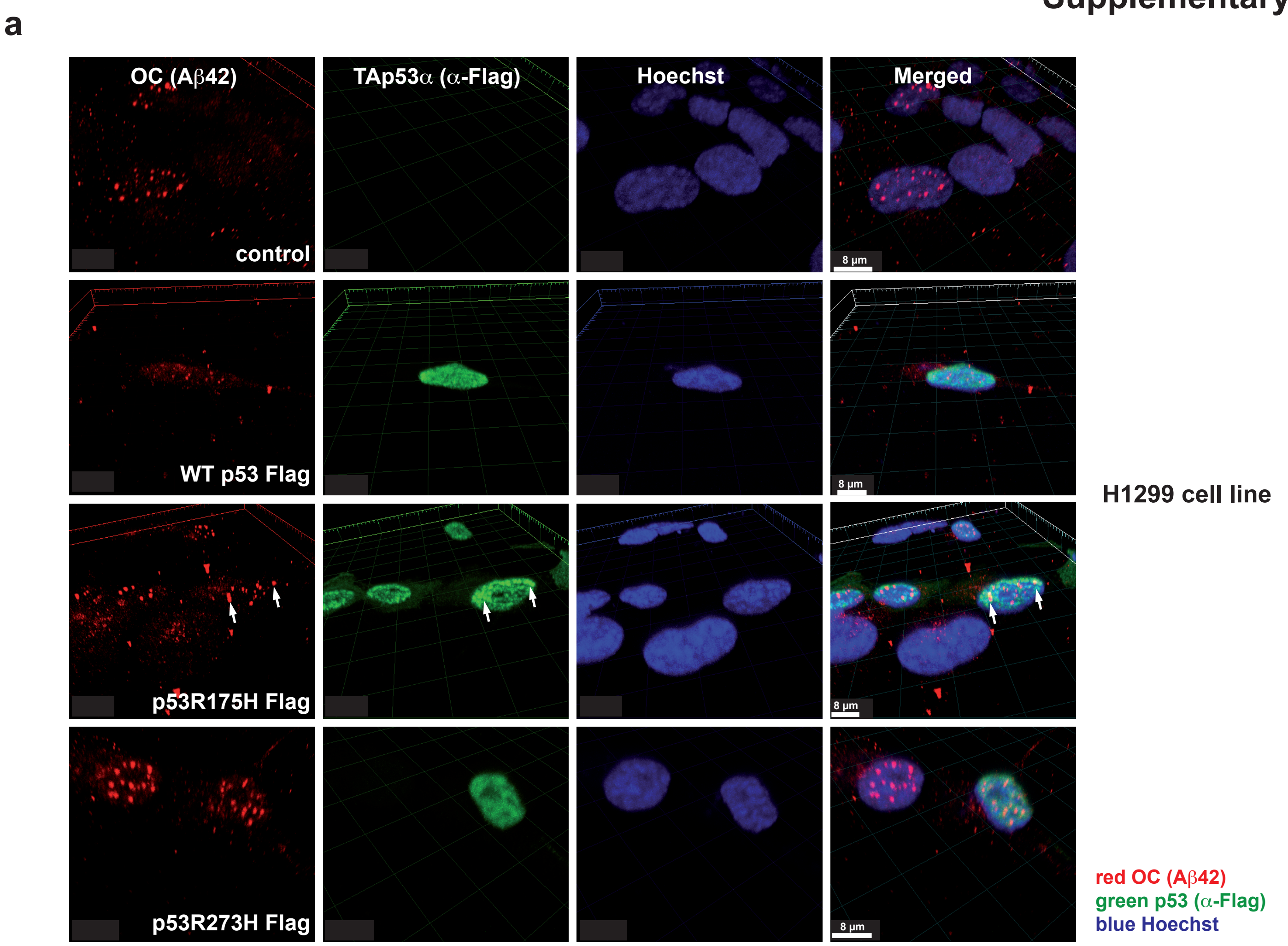

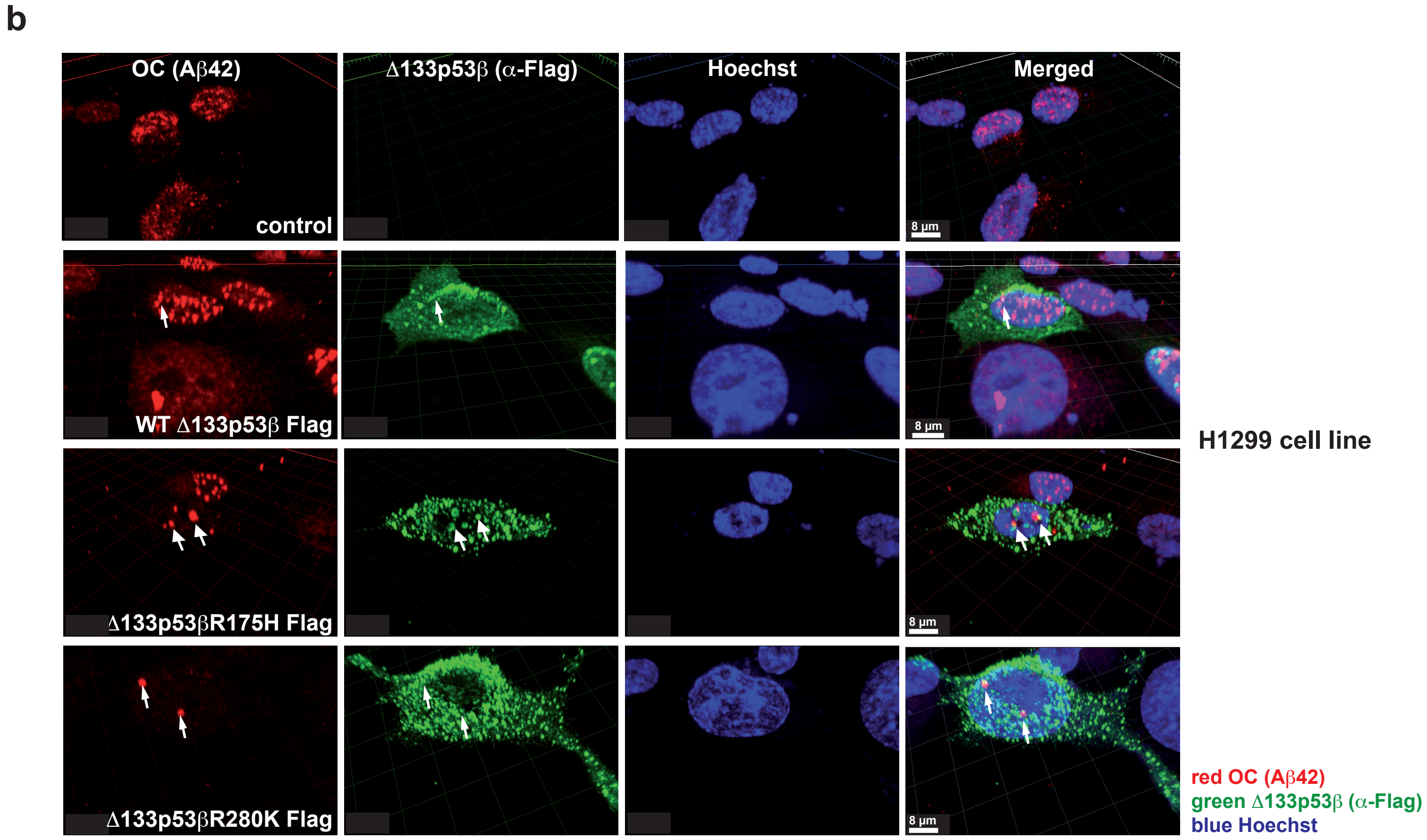

Supplementary Figure 1

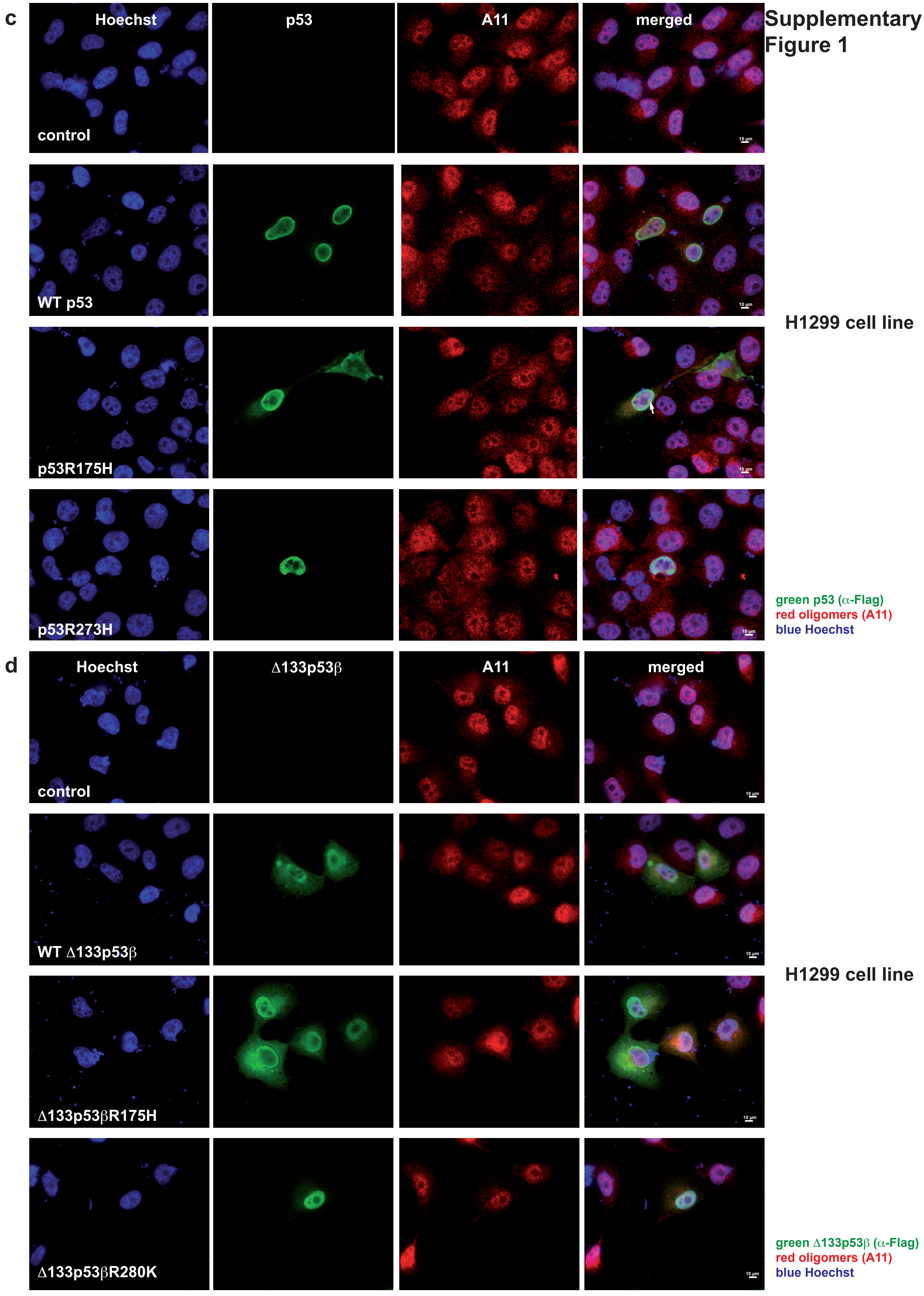

Supplementary Figure 1. Immunofluorescent analysis p53 isoforms co-localisation with amyloid aggregates and protein oligomers.

a. 3D reconstruction of confocal immunofluorescent Z-stack images of H1299 cells transfected with WT, structurally and contact mutated p53 with separated acquisition channels. Green: α-Flag (p53), red: α-OC (Aβ42, amyloid aggregates) antibody, blue: Hoechst. Scale bar 8μm. n=3

b. 3D reconstruction of the confocal immunofluorescent Z-stack images of H1299 cells transfected with WT, structurally and contact mutated Δ133p53β with separated acquisition channels. Green: α-Flag (Δ133p53β), red: α-OC (Aβ42, amyloid aggregates) antibody, blue: Hoechst. Scale bar 8μm. n=3

c. Immunofluorescent analysis wt, structural and contact mutant of p53 co-localisation with protein oligomers visualised by A11 antibody. Green: Flag (p53), Red: protein oligomers (A11 antibody), Blue: Hoechst. n=3

d. Immunofluorescent analysis wt, structural and contact mutant of Δ 133p53β co-localisation with protein oligomers visualised by A11 antibody. Green: Flag (Δ133p53β), Red: protein oligomers (A11 antibody), Blue: Hoechst. n=3

The arrows indicate co-localisation.

Scale bar 10μm.

Source data are provided as a Source Data file.

Supplementary Figure 2

a

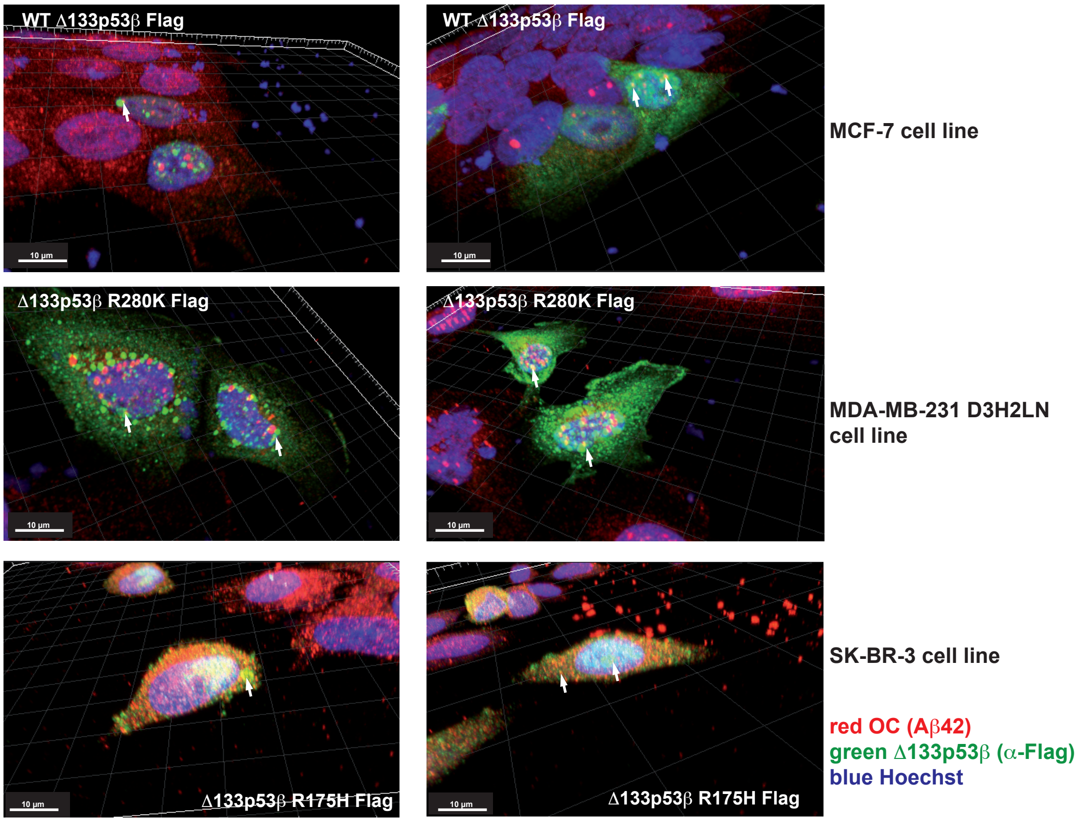

b

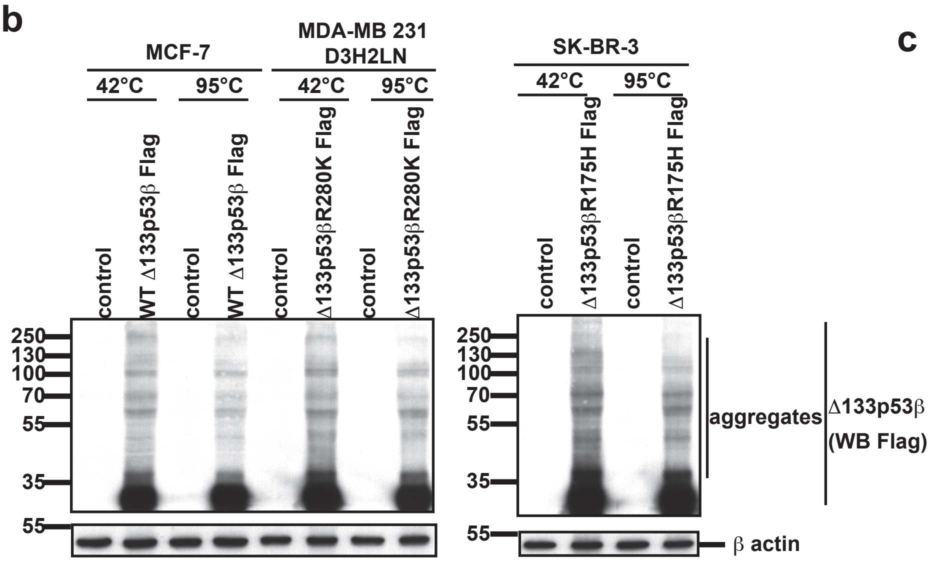

c

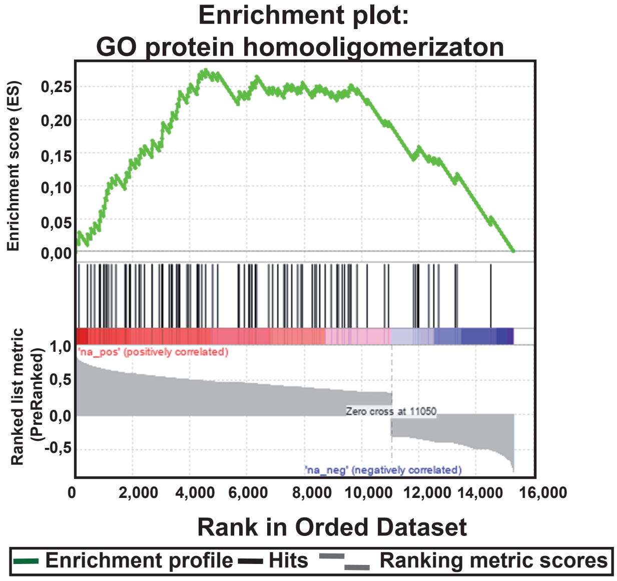

d

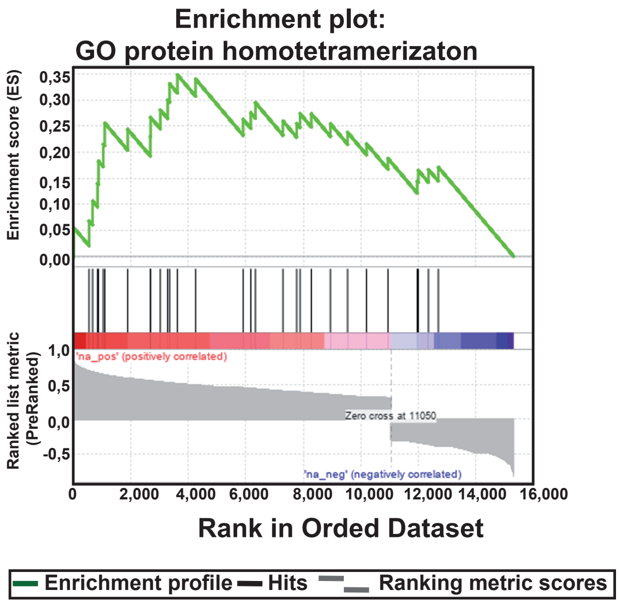

e

Homo tetramerization	Δ133p53β correlation
KCNJ12	0.522
RNF135	0.511
KCTD5	0.483
FUS	0.476
TRPM2	0.448
CRTC2	0.399
KCTD15	0.392
POLQ	0.344
ALDOA	0.329
BLM	0.328
MCOLN1	0.308

Supplementary Figure 2. Evaluation of aggregate forming capacity of Δ133p53β isoform in different breast cancer cell lines.

a. Immunofluorescent analysis of Δ133p53β co-localisation with amyloid type aggregates in MCF-7, MDA-MB 231 D3H2LN and SK-BR-3 cells upon 3D reconstruction of confocal Z-stacks. Green: α-Flag (Δ133p53β), Red: α-OC (Aβ42, amyloid aggregates), blue: Hoechst. Scale bar 10 µm. n=3

b. Western blot analysis of Δ133p53β aggregation forming capacity in MCF-7, MDA-MB 231 D3H2LN and SK-BR-3 cells from the soluble protein fraction. n=3

c. Enrichment plot obtained by Gene Ontology analysis for protein homoligomerisarion processes upon RNAseq analysis performed on prostate cancer samples that correlate with Δ133p53β expression. n=3

d. Enrichment plot obtained by Gene Ontology analysis for protein homotetramerisation processes upon RNAseq analysis performed on prostate cancer samples that correlate with Δ133p53β expression.

e. Examples of genes involved in cellular processes linked to tetramerization obtained by Gene Ontology analysis for protein homotetramerisation processes upon RNAseq analysis performed on prostate cancer samples that correlate with Δ133p53β expression.

The arrows indicate co-localisation.

Molecular weight is expressed in kDa.

Source data are provided as a Source Data file.

Supplementary Figure 3

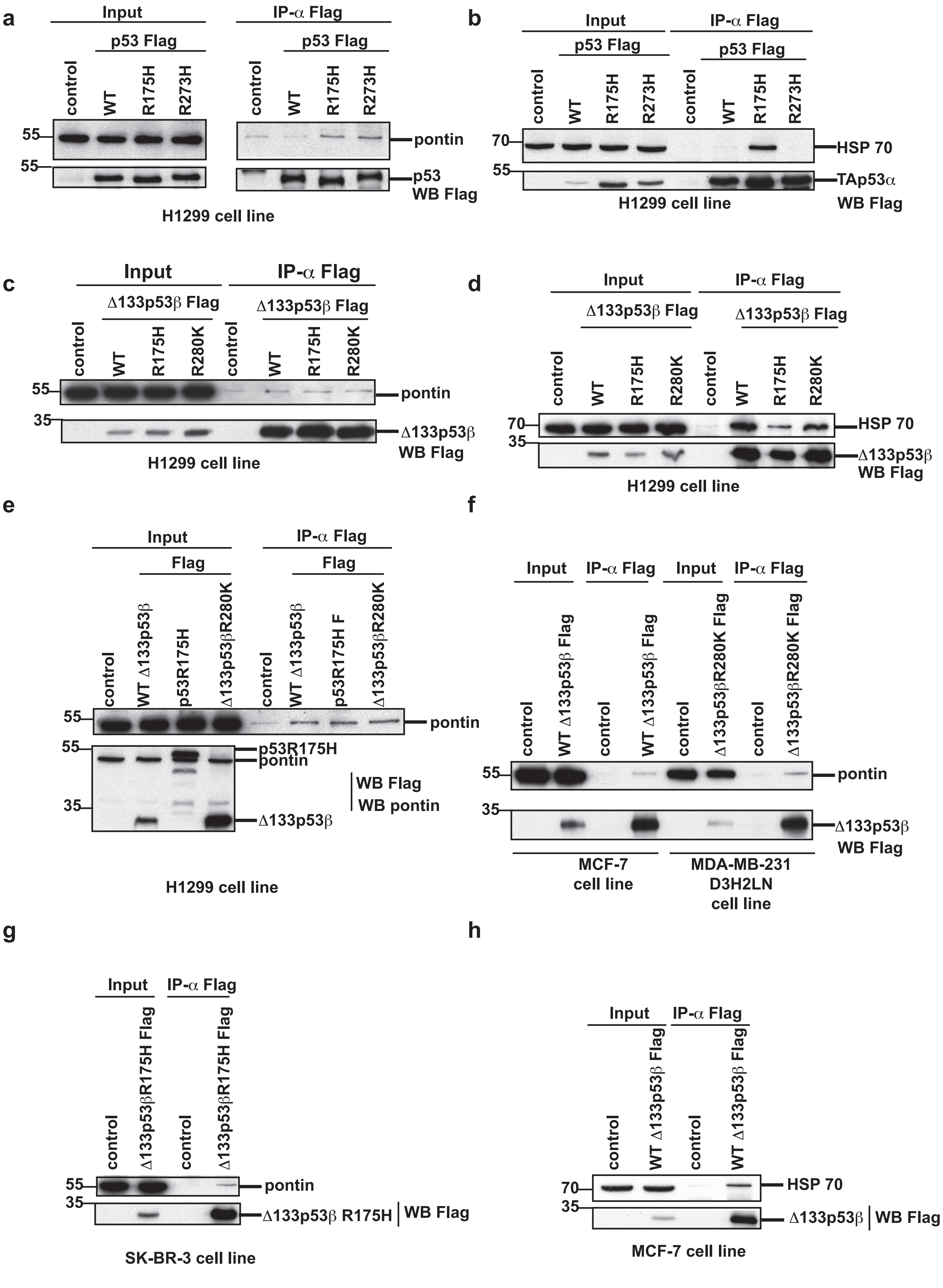

Supplementary Figure 3

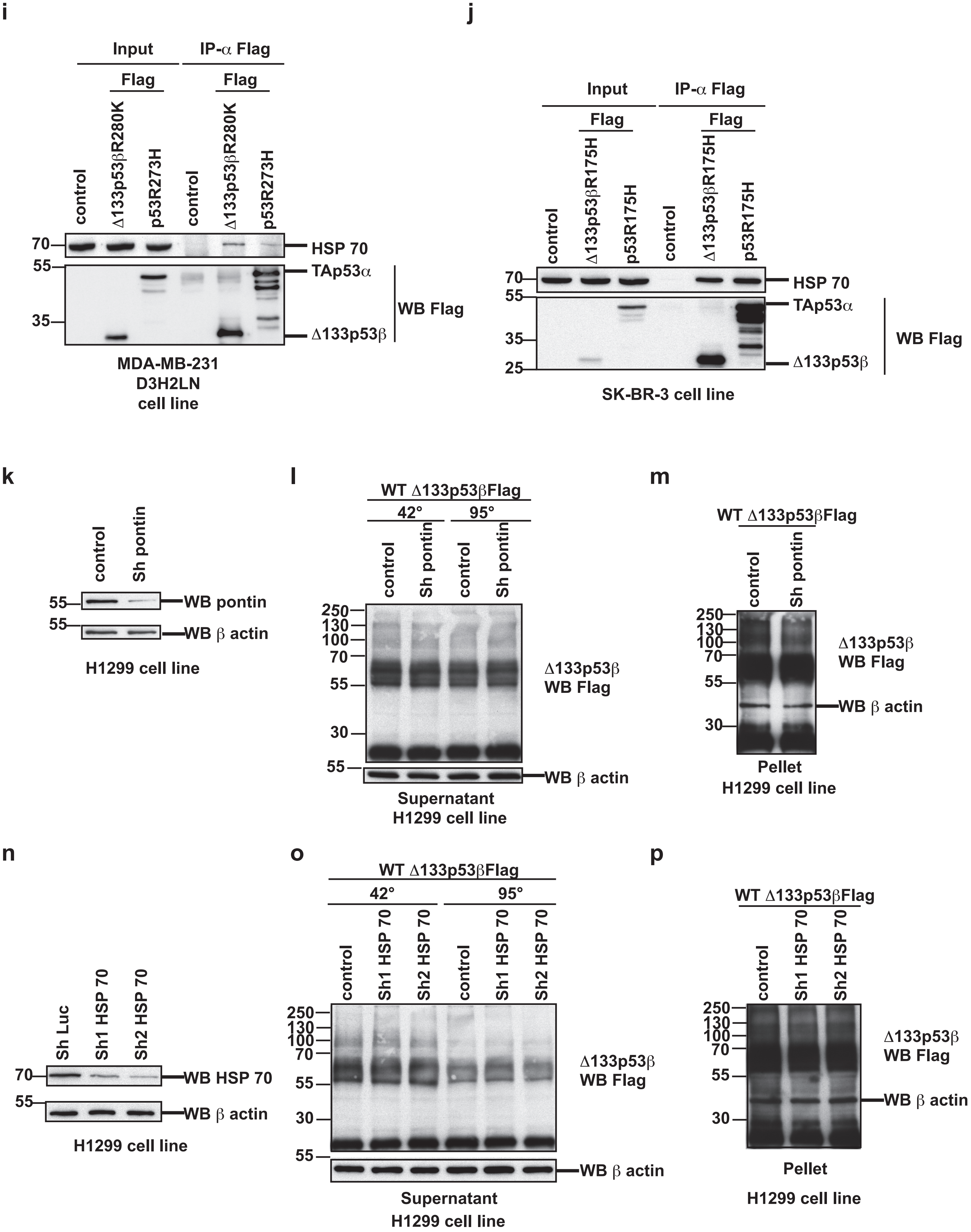

Supplementary Figure 3. Evaluation of the Pontin and HSP70 effects on Δ133p53β aggregation capacity.

a. Co-immunoprecipitation analysis of WT p53, structurally and contact mutated proteins association with Pontin in H1299 cell line. n=3

b. Co-immunoprecipitation analysis of WT p53, structurally and contact mutated proteins association with HSP70 in H1299 cell line. n=5

c. Co-immunoprecipitation analysis of WT Δ133p53β, structurally and contact mutated proteins association with Pontin in H1299 cell line. n=3

d. Co-immunoprecipitation analysis of WT Δ133p53β, structurally and contact mutated proteins association with HSP70 in H1299 cell line. n=5

e. Co-immunoprecipitation analysis of Pontin interaction with different p53 isoforms upon expression in H1299 cells. n=3

f and **g.** Co-immunoprecipitation analysis of Pontin interaction with Δ133p53β isoform in MCF-7, MDA-MB 231 D3H2LN and SK-BR-3 cells. n=3

h-j. Co-immunoprecipitation analysis of HSP70 interaction with Δ133p53β isoform in MCF-7, MDA-MB 231 D3H2LN and SK-BR-3 cells. n=5

k. Western blot analysis of Pontin depletion upon Sh RNA application in H1299 cell line. n=2

l. Western blot analysis of the soluble protein fraction from H1299 cells after Δ133p53β transfection in control and Pontin depleted backgrounds. n=2

m. Western blot analysis of the insoluble protein fraction from H1299 cells after WT Δ133p53β transfection in control and Pontin depleted backgrounds.n=2

n. Western blot analysis of HSP70 depletion upon two different Sh RNA applications in H1299 cell line. n=3

o. Western blot analysis of the soluble protein fraction from H1299 cells after Δ133p53β transfection in control and HSP70 depleted backgrounds. n=3

p. Western blot analysis of the insoluble protein fraction from H1299 cells after WT Δ133p53β transfection in control and HSP70 depleted backgrounds. n=3

Molecular weight is expressed in kDa.

Source data are provided as a Source Data file.

Supplementary Figure 4

a

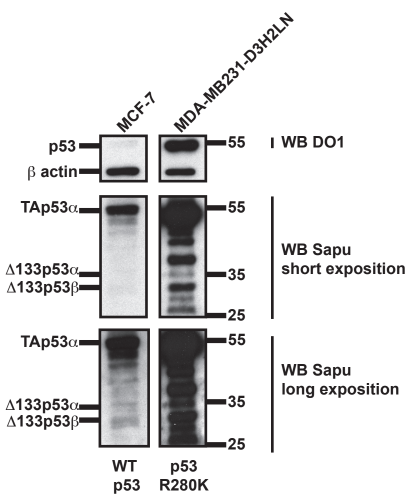

b

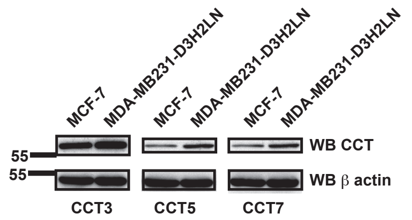

Supplementary Figure 4. **p53 isoform and CCT complex abundance in MCF-7 and MDA-MB-231 D3H2LN.**

a. Western blot analysis of p53 isoform abundance in MCF-7 and MDA-MB-231 D3H2LN cell lines. n=3

b. Western blot analysis of CCT complex subunit abundance in MCF-7 and MDA-MB-231 D3H2LN cell lines. n=3

Molecular weight is expressed in kDa.

Source data are provided as a Source Data file.

Supplementary Figure 5

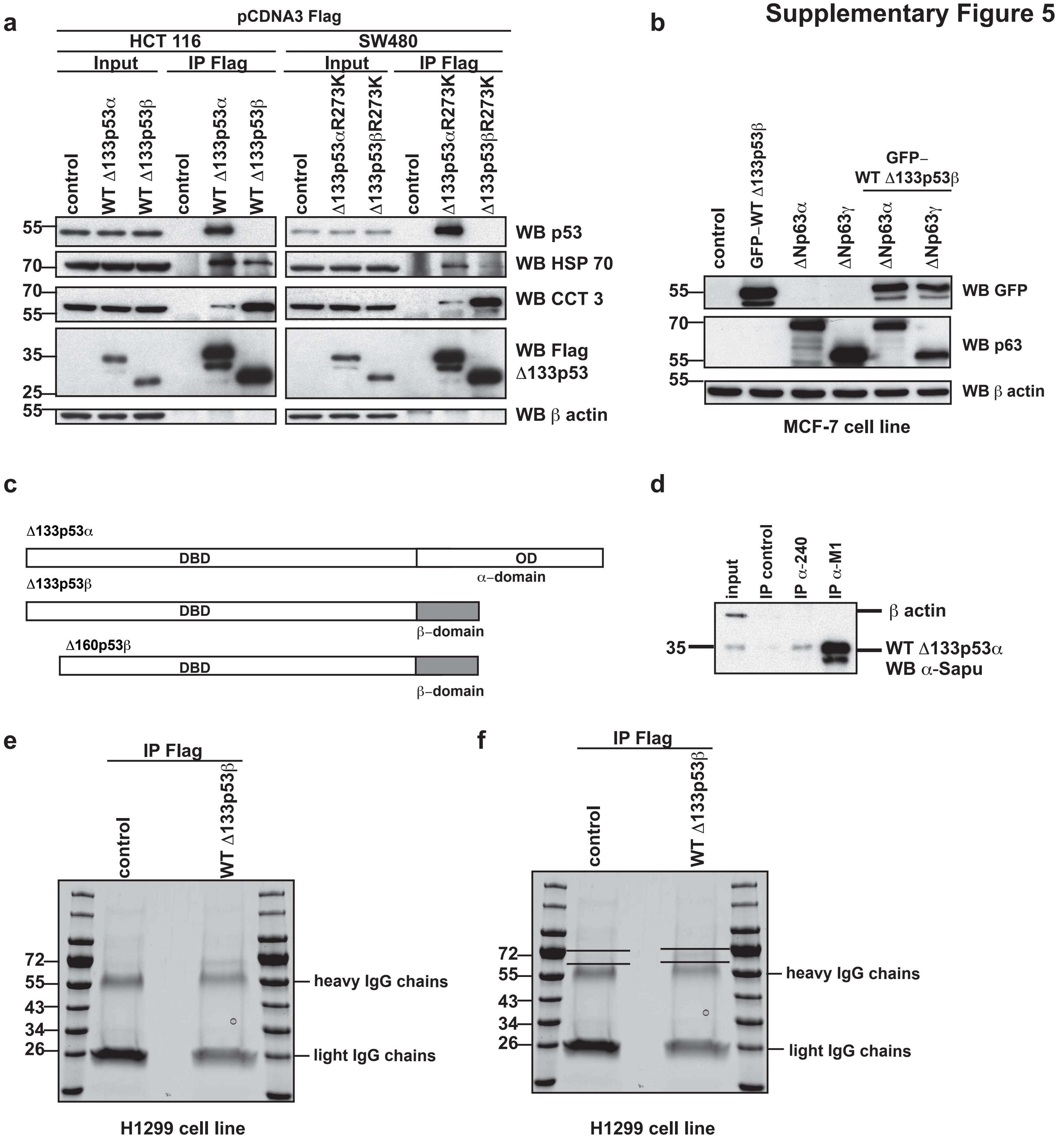

Supplementary Figure 5. Co-immunoprecipitation and expression analysis of WT Δ133p53β interactions in different cellular backgrounds.

a. Co-immunoprecipitation analysis of WT Δ133p53α and WT Δ133p53β interactions with different partners upon expression in HCT116 and SW480 colon cancer cell lines.
n=3-5 for different panels of the figure
b. Western blot analysis of expression and co-expression of EGFP tagged WT Δ133p53β and ΔNp63α and ΔNp63γ isoforms used for time-lapse imaging (Supplementary Movies 4-9). Representative immunoblot of one out of 3 experiments is shown.
c. Schematic representation of Δ133p53α, β and Δ160p53β isoforms.
d. Immunoprecipitation analysis of WT Δ133p53α isoform by Ab 240 and CM1 in H1299 cell line. n=2
e. Silver stained polyacrylamide gel upon electrophoretic separation of immunoprecipitation reaction with α-Flag beads performed on control and Δ133p53β transfected H1299 cells. In total 3 different proteomic analyses were performed. The most pertinent is shown.
f. Delineated regions of the silver stained polyacrylamide gel upon electrophoretic separation of immunoprecipitation reaction with α-Flag beads performed on control and Δ133p53β transfected H1299 cells that were dissected and used for proteomic analysis.
In total 3 different proteomic analyses were performed. The most pertinent is shown.
Molecular weight is expressed in kDa.

**Tissue type**			**n**	**% of samples with aggregates**	**% of cells with aggregates**
**Breast tumour**	Brain metastasis	Triple negative	5	100%	11, 30, 55, 70, and 77%
	Brain metastasis	Her2 positive	5	80% (n=4)	12, 15*, 19 and 25%
	Primary	Triple negative	5	60% (n=3)	18, 27*, and 30%*
	Primary	Her2 positive	5	40% (n=2)	17* and 26%
	Primary	ER+PR+ HER2-	5	20% (n=1)	Scattered positive
**Breast normal associated**			15	20% (n=3)	Scattered positive
**Lung tumour**	Brain metastasis	Adenocarcinoma	5	40% (n=2)	9* and 25%*
	Primary	Adenocarcinoma	3	0%	
**Lung normal associated**			3	0%	
**Colorectal tumour**	Brain metastasis	Adenocarcinoma	3	67% (n=2)	8* and 12%*

*, aggregates present in parts of the tumour only. In these tumours the percent of cells with aggregates were counted in positive areas

Supplementary Table 1. Quantification of aggregates in human tumour samples

Target gene	Sequence
Pontin	GCATGACTTGGATGTGGCTAATGC
HSP70	CGTGGAGGAGTTCAAGAGAA
HSP70	GCCATGACGAAAGACAACAA
ΔNp63	GGACAGCAGCATTGATCAA
TAp63	TTCCTCAGTCCAGAGGTTT
Δ133p53	GGAGGTGCTTACACATGTT
CCT2	GCCTCTCTTATGGTAACCAA
CCT3	GCCAAGTCCATGATCGAAAT
CCT5	CCACTTCTGTGATTAAGTA
CCT7	GCCACAATTCTGAAACTTCT
SiΔ133-1	GGAGGTGCTTACACATGTT
SiΔ133-2	CTTGTGCCCTGACTTTCAA
Siβ	GGACCAGACCAGCTTTCA

Supplementary Table 2. List of Sh and Si RNA used for gene silencing

Antibody	Reference
Flag	mouse monoclonal 1804, Sigma
p53	Ab 240, mouse monoclonal [1]
p53	DO-1, mouse monoclonal sc-126, Santa Cruz Biotechnology
p53	Sapu, sheep polyclonal p53 pantropic [2]
p53	CM1, rabbit polyclonal [3]
p53	PAb421 antibody, Millipore Sigma, Burlington, MA, USA),
Pontin	RUVBL1 rabbit polyclonal ref 102-10-2-AP, Proteintech,
HSP70	mouse monoclonal (W27):sc-24 Santa Cruz Biotechnology
CCT2	rat monoclonal VMA00026 Bio-Rad
CCT3	goat polyclonal VPA00113 Bio-Rad
CCT5	rat monoclonal MCA2178 Bio-Rad
CCT7	rat monoclonal MCA2179 Bio-Rad
GFP	rabbit polyclonal anti GFP (Invitrogen, Life Technologies, A-6455)
p63	rabbit monoclonal Abcam, Ab 124762
p63	mouse monoclonal Abnova, MAB7941 clone 4A4
E cadherin	mouse monoclonal Cat No 131700 Zymed (now Life Technologies)
N cadherin	mouse monoclonal Cat No. 610921 BD Biosciences
tubulin	mouse monoclonal T6199 Sigma
β actin	mouse monoclonal A5441 Sigma
β p53 KJC8	(Bourdon et al., 2005)
Oligomer A11 antibody	Rabbit polyclonal Invitrogen AHB0052
α-p63	SFI-6 antibody (DCS Innovative Diagnostik-Systeme Germany)

Supplementary Table 3. List of the antibodies used for immunoblot and immunofluorescent analysis

Antibody	dilution
α-p53 DO-1	1:500
α-pontin	1:250
α-Flag	1:500
α-p53 Sapu,	1:6000
α-β actin	1:10000
α-HSP70	1:500
α-CCT2, 3, 5 and 7	1:1000
α-p63	1:1000
α-E cadherin	1:500
α-N cadherin	1:500
α-tubulin	1:10000
α-GFP	1:500

Supplementary Table 4. Dilutions of the antibodies used for immunoblots

Antibody	dilution
α-Flag	1:500
α-Amyloid Fibrils OC	1:500
Oligomer A11 antibody	1:500
α- p63 (Abcam, Ab 124762)	1:500
KJC8	1:50
PAb421	1:100

Supplementary Table 5. Dilutions of the antibodies used for immunofluorescent analysis

exon	name	sequence
E3	TAp63(e3)f	TTAGCATGGACTGTATCCGC
E3'	DelNp63F	GCCCAGACTCAATTTAGTGAG
E14	RT2p63	GCTCAGGGATTTTCAGACTTGCCAGATC
E10'	p63grt1	GCTCCACAAGCTCATTCCTG
E10b	p63epsr3	CACTCATGCCTCCTAAAATGACA
E13-E14	p63alpharev	ACTTGCCAGATCATCCATGG
E12-14	p63betaR	GCCAGATCCTGACAATGCTG
E11-E14	p63delta rev	ACTTGCCAGATCTGTTGG
E10'	p63gamma rev	CTCATTCCTGAAGCAGGCTG
E10b	RTp63eps2	GCCTCCTAAAATGACACGTTGATAC

Supplementary Table 6. List of the oligos used for p63 isoforms detection by nested PCR

<table>
<tr><th></th><th colspan="2">1st PCR</th><th colspan="2">2nd PCR</th></tr>
<tr><td>TAp63 α</td><td rowspan="5">TAp63 e3f</td><td rowspan="3">RT2p63</td><td rowspan="5">TAp63 e3f</td><td>p63 α rev</td></tr>
<tr><td>TAp63 β</td><td>p63 β rev</td></tr>
<tr><td>TAp63 δ</td><td>p63 δ rev</td></tr>
<tr><td>TAp63 γ</td><td>p63 grt1</td><td>p63 γ rev</td></tr>
<tr><td>TAp63 ε</td><td>p63 epsr3</td><td>RTp63 eps2</td></tr>
<tr><td>ΔNp63 α</td><td rowspan="5">ΔNp63 f</td><td rowspan="3">RT2p63</td><td rowspan="5">ΔNp63 f</td><td>p63 α rev</td></tr>
<tr><td>ΔNp63 β</td><td>p63 β rev</td></tr>
<tr><td>ΔNp63 δ</td><td>p63 δ rev</td></tr>
<tr><td>ΔNp63 γ</td><td>p63 g rt1</td><td>p63 γ rev</td></tr>
<tr><td>ΔNp63 ε</td><td>p63 epsr3</td><td>RTp63 eps2</td></tr>
</table>

Supplementary Table 7. List of the oligos used for 1st and 2nd PCR reaction for p63 isoforms detection

Tumour type	Tumour details	No of patients	Sex	Age	Treatment
Breast metastases to the brain	-Triple negative<br><br>-Her2 positive	5<br><br>5	Female	40-76	-Paclitaxel, vinorelbine, and radiotherapy (3 patients)<br>-Paclitaxel and Letrozole (2 patients)<br>-Docetaxel, vinorelbine and Herceptin radiotherapy (4 patients)<br>-Herceptin (1 patient)
Primary breast tumour	-Triple negative<br><br>-Her2 positive<br><br>-ER and PR positive, and Her2 negative	5<br><br>5<br><br>5	Female	41-80	-Paclitaxel, vinorelbine, and radiotherapy (3 patients)<br>-Paclitaxel and vinorelbine (1 patient)<br>-Vinorelbine, and radiotherapy ((1 patient)<br>-Docetaxel, herceptin, and radiotherapy (3 patients)<br>-Docetaxel and Herceptin (1 patient)<br>-Docetaxel, vinorelbine, and Herceptin (1 patient)<br>-Tamoxifen and radiotherapy (3 patients)<br>Tamoxifen, letrozole, and radiotherapy (1 patient)<br>Tamoxifen and letrozole (1 patient)
Lung metastases to the brain	Metastatic adenocarcinoma	5	Male (2 patients)<br>Female (3 patients)	39-71	-Radiotherapy (3 patients)<br>-Gefitinib, radiotherapy (1 patient)<br>Radiotherapy, Cisplatin, and vinblastine (1 patient)
Lung primary	Adenocarcinoma	3	Male (2 patients)<br>Female (1 patient)	50-61	-Radiotherapy, cisplatin, and vinblastine (2 patients)<br>-Radiotherapy and gefitinib (1 patient)
Colorectal metastases to the brain	Metastatic adenocarcinoma Bowel origin	3	Male (2 patients)<br>Female (1 patient)	54-58	-FOLFOX, FOLFIRI with cetuximab (2 patients)<br>- FOLFOX, FOLFIRI with radiotherapy (1 patient)

Supplementary Table 8: Clinical characteristics of the analysed patients.

Supplementary references:

1 Yewdell, J. W., Gannon, J. V. & Lane, D. P. Monoclonal antibody analysis of p53 expression in normal and transformed cells. *Journal of virology* **59**, 444-452, doi:10.1128/jvi.59.2.444-452.1986 (1986).

2 Vojtesek, B. *et al.* Conformational changes in p53 analysed using new antibodies to the core DNA binding domain of the protein. *Oncogene* **10**, 389-393 (1995).

3 Midgley, C. A. *et al.* Analysis of p53 expression in human tumours: an antibody raised against human p53 expressed in Escherichia coli. *Journal of cell science* **101 (Pt 1)**, 183-189 (1992).
