# Supplementary material for: Δ133p53β isoform pro-invasive activity is regulated through an aggregation-dependent mechanism in cancer cells
Source: Nat Commun. 2021 Sep 15;12:5463. doi: 10.1038/s41467-021-25550-2 (PMC8443592; doi:10.1038/s41467-021-25550-2)
Supplement: Supplementary file 3 — Description of Additional Supplementary Files [file 41467_2021_25550_MOESM3_ESM.pdf]

Description of Additional Supplementary Files

File Name: Supplementary Movie 1
Description: Time-lapse microscopy of pWTΔ133p53βEGFP transfected H1299 cells 5-40h post-transfection.

File Name: Supplementary Movie 2
Description: Time-lapse microscopy of pcDNA3mCherryΔNp63α transfected H1299 cells 5-40h post-transfection.

File Name: Supplementary Movie 3
Description: Time-lapse microscopy of pWTΔ133p53βEGFP and pcDNA3mCherry ΔNp63α transfected H1299 cells 5-40h post-transfection.

File Name: Supplementary Movie 4
Description: DIC time-lapse microscopy of mock transfected MCF-7 cells 4-20h post-transfection.

File Name: Supplementary Movie 5
Description: DIC time-lapse microscopy of EGFP-WTΔ133p53β transfected MCF-7 cells 4-20h post-transfection.

File Name: Supplementary Movie 6
Description: DIC time-lapse microscopy of ΔNp63α transfected MCF-7 cells 4-20h post-transfection.

File Name: Supplementary Movie 7
Description: DIC time-lapse microscopy of ΔNp63γ transfected MCF-7 cell line 4-20h post-transfection.

File Name: Supplementary Movie 8
Description: DIC time-lapse microscopy of MCF-7 cells co-expressing EGFP-WT Δ133p53β and ΔNp63α 4-20h post-transfection.

File Name: Supplementary Movie 9
Description: DIC time-lapse microscopy of MCF-7 cells co-expressing EGFP-WT Δ133p53β and ΔNp63γ 4-20h post-transfection.

File Name: Supplementary Movie 10
Description: Time-lapse microscopy of pEGFP transfected H1299 cells during FRAP experiment.

File Name: Supplementary Movie 11
Description: Time-lapse microscopy of pWTΔ133p53βEGFP transfected H1299 cells during FRAP experiment.

File Name: Supplementary Movie 12
Description: Time-lapse microscopy of pWTΔ133p53βEGFP and pcDNA3mCherry ΔNp63α transfected H1299 cells during FRAP experiment.
