# Supplementary material for: Δ133p53β isoform pro-invasive activity is regulated through an aggregation-dependent mechanism in cancer cells
Source: Nat Commun. 2021 Sep 15;12:5463. doi: 10.1038/s41467-021-25550-2 (PMC8443592; doi:10.1038/s41467-021-25550-2)
Supplement: Supplementary file 16 — Reporting Summary [file 41467_2021_25550_MOESM16_ESM.pdf]

nature portfolio

Corresponding author(s): Pierre Roux
Manuscript NCOMMS-19-40469-T

Last updated by author(s): Jun 9, 2021

Reporting Summary

Nature Portfolio wishes to improve the reproducibility of the work that we publish. This form provides structure for consistency and transparency in reporting. For further information on Nature Portfolio policies, see our Editorial Policies and the Editorial Policy Checklist.

Statistics

For all statistical analyses, confirm that the following items are present in the figure legend, table legend, main text, or Methods section.

n/a	Confirmed	
☐	☒	The exact sample size (n) for each experimental group/condition, given as a discrete number and unit of measurement
☐	☒	A statement on whether measurements were taken from distinct samples or whether the same sample was measured repeatedly
☐	☒	The statistical test(s) used AND whether they are one- or two-sided<br>*Only common tests should be described solely by name; describe more complex techniques in the Methods section.*
☒	☐	A description of all covariates tested
☒	☐	A description of any assumptions or corrections, such as tests of normality and adjustment for multiple comparisons
☐	☒	A full description of the statistical parameters including central tendency (e.g. means) or other basic estimates (e.g. regression coefficient) AND variation (e.g. standard deviation) or associated estimates of uncertainty (e.g. confidence intervals)
☒	☐	For null hypothesis testing, the test statistic (e.g. F, t, r) with confidence intervals, effect sizes, degrees of freedom and P value noted<br>*Give P values as exact values whenever suitable.*
☒	☐	For Bayesian analysis, information on the choice of priors and Markov chain Monte Carlo settings
☒	☐	For hierarchical and complex designs, identification of the appropriate level for tests and full reporting of outcomes
☒	☐	Estimates of effect sizes (e.g. Cohen's d, Pearson's r), indicating how they were calculated

Our web collection on statistics for biologists contains articles on many of the points above.

Software and code

Policy information about availability of computer code

Data collection	*Provide a description of all commercial, open source and custom code used to collect the data in this study, specifying the version used OR state that no software was used.*
Data analysis	About availablity to check our results by using software mentioned in the manuscript. Below are the web-interfaces for these software:<br>1. IUpred online tool (http://iupred.elte.hu/)<br>2. CMView can be downloaded from http://www.bioinformatics.org/cmview/download.html<br>3. ArchCandy program can be downloaded from https://www.dropbox.com/s/6r7szv91vvdv364/ArchCandy_01.09.2014.jar?dl=0<br>4. Waltz online tool - http://waltz.switchlab.org/<br>5. PASTA online tool - http://protein.bio.unipd.it/pasta2/

For manuscripts utilizing custom algorithms or software that are central to the research but not yet described in published literature, software must be made available to editors and reviewers. We strongly encourage code deposition in a community repository (e.g. GitHub). See the Nature Portfolio guidelines for submitting code & software for further information.

March 2021

Data

Policy information about availability of data

All manuscripts must include a data availability statement. This statement should provide the following information, where applicable:

- Accession codes, unique identifiers, or web links for publicly available datasets
- A description of any restrictions on data availability
- For clinical datasets or third party data, please ensure that the statement adheres to our policy

Data Availability
The mass spectrometry proteomics data have been deposited to the ProteomeXchange Consortium via the PRIDE (Perez-Riverol et al., 2019) partner repository with the dataset identifier PXD022998. Previously published RNA-sequencing data has been used for gene set enrichment analysis (Kazantseva et al., 2019).
The Figures 1A, 1B,1C, 1E, 1F, 1G, 1H, 1I, Figures 2A, 2B, 2C, 2E, 2F, 2G, Figures 3A, 3B, 3C, 3D, 3E, 3F, 3G, 3H, 3I, Figures 4A, 4A, 4C, 4D, 4E, 4F, 4G, Figures 5A, 5B, 5C, 5D, 5E, 5F, 5G, 5H, 5I, Figures 6A, 6B, 6C, 6D, 6E, Supplementary figures 1A, 1B, 1C, 1D, Supplementary figures 2A, 2B, 2C, 2D, Supplementary figures 3A, 3B, 3C, 3D, 3E, 3F, 3G, 3H, 3I, 3J, 3K, 3L, 3M, 3N, 3O, 3P, Supplementary figures 4A, 4B, Supplementary figures 5A, 5B, 5C, 5D, 5E, 5F data generated in this study are provided as a Source Data file.
Source data are provided with this paper.

Field-specific reporting

Please select the one below that is the best fit for your research. If you are not sure, read the appropriate sections before making your selection.

☒ Life sciences ☐ Behavioural & social sciences ☐ Ecological, evolutionary & environmental sciences

For a reference copy of the document with all sections, see nature.com/documents/nr-reporting-summary-flat.pdf

Life sciences study design

All studies must disclose on these points even when the disclosure is negative.

Sample size	Triplicates or quadruplicate were used as a minimum number of independent replicates, as a typical
Data exclusions	Data were excluded only in cases where controls samples were not appropriate, for example not efficient knockdown for the gene of interest, unequal total protein loading. These however represent minor instances.
Replication	Triplicates were used as a minimum number of independent replicates.
Randomization	In immunofluorescence experiments, for each condition at least 75 cells were counted in 3 or 4 randomly selected imaging areas.
Blinding	Blinding in not a common practice in this area of molecular/cellular biology and was not applied in this study

Reporting for specific materials, systems and methods

We require information from authors about some types of materials, experimental systems and methods used in many studies. Here, indicate whether each material, system or method listed is relevant to your study. If you are not sure if a list item applies to your research, read the appropriate section before selecting a response.

Materials & experimental systems

n/a	Involved in the study	
☐	☒	Antibodies
☐	☒	Eukaryotic cell lines
☐	☐	Palaeontology and archaeology
☐	☒	Animals and other organisms
☐	☒	Human research participants
☒	☐	Clinical data
☒	☐	Dual use research of concern

Methods

n/a	Involved in the study	
☒	☐	ChIP-seq
☒	☐	Flow cytometry
☒	☐	MRI-based neuroimaging

Antibodies

Antibodies used	All antibodies (including clones, catalogue numbers) are described in Methods. The lot number was not always available in the appropriate websites

Validation	Validation was relied on the available data on commercial websites and previous studies which have performed knockdown experiments. Is some cases we performed knockdown experiments to validate the used antibodies.

Eukaryotic cell lines

Policy information about cell lines

Cell line source(s)	Unless otherwise stated (gift from another laboratory), the American Type Culture Collection was the original source of used cell lines.
Authentication	None of the cell lines were authenticated. This is stated in the Methods
Mycoplasma contamination	Cells were routinely tested for mycoplasma contamination. This is stated in Methods
Commonly misidentified lines (See ICLAC register)	NA

Palaeontology and Archaeology

Specimen provenance	*Provide provenance information for specimens and describe permits that were obtained for the work (including the name of the issuing authority, the date of issue, and any identifying information). Permits should encompass collection and, where applicable, export.*
Specimen deposition	*Indicate where the specimens have been deposited to permit free access by other researchers.*
Dating methods	*If new dates are provided, describe how they were obtained (e.g. collection, storage, sample pretreatment and measurement), where they were obtained (i.e. lab name), the calibration program and the protocol for quality assurance OR state that no new dates are provided.*

☐ Tick this box to confirm that the raw and calibrated dates are available in the paper or in Supplementary Information.

Ethics oversight	*Identify the organization(s) that approved or provided guidance on the study protocol, OR state that no ethical approval or guidance was required and explain why not.*

Note that full information on the approval of the study protocol must also be provided in the manuscript.

Animals and other organisms

Policy information about studies involving animals; ARRIVE guidelines recommended for reporting animal research

Laboratory animals	Species is Mus musculus, strain C57B6, homozygote D122p53 male mice, 7 weeks of age.
Wild animals	*Provide details on animals observed in or captured in the field; report species, sex and age where possible. Describe how animals were caught and transported and what happened to captive animals after the study (if killed, explain why and describe method; if released, say where and when) OR state that the study did not involve wild animals.*
Field-collected samples	*For laboratory work with field-collected samples, describe all relevant parameters such as housing, maintenance, temperature, photoperiod and end-of-experiment protocol OR state that the study did not involve samples collected from the field.*
Ethics oversight	Mouse studies were approved by the University of Otago Animal Ethics Committee - AEC 03/12.

Note that full information on the approval of the study protocol must also be provided in the manuscript.

Human research participants

Policy information about studies involving human research participants

Population characteristics	we have added a table (table 8) describing the clinical characteristics of the patients analyzed, as requested in the editing process.In this table are described age, gender and genotypic information (hormonal and EGFR status for breast tumours, TMN stage for the primary lung cancers). Current diagnosis is given and previous diagnosis is inferred for these cases: for example brain tumour metastasis from the lung will have a previous diagnosis of primary lung cancer.
Recruitment	A prospective collection of tumors from patients who agreed to participate in the study was already available. Primary breast, lung and colorectal tumors as well as the corresponding brain metastases were first tested for the expression of Δ133p53β mRNA by RNAscope and then, positive tumors were tested for the presence of aggregates. For breast tumors, up to five cases for each of the different receptor statuses were analyzed.<br>The ability of tumors to form Δ133p53β aggregates was therefore carried out on tumors selected to express Δ133p53β. There is thus no bias due to the selection of tumors with or without aggregates, which was the objective of the study
Ethics oversight	Ethical approval for work using human tumours (reference LRS/10/09/037 and MEC/08/02/061) was obtained in New Zealand and all procedures followed institutional guidelines. All individuals provided written informed consent.

Note that full information on the approval of the study protocol must also be provided in the manuscript.
